# Mesenchymal Stem Cell Protection of Neurons against Glutamate Excitotoxicity Involves Reduction of NMDA-Triggered Calcium Responses and Surface GluR1, and Is Partly Mediated by TNF

**DOI:** 10.3390/ijms19030651

**Published:** 2018-02-25

**Authors:** Irini Papazian, Vasiliki Kyrargyri, Maria Evangelidou, Anda Voulgari-Kokota, Lesley Probert

**Affiliations:** 1 Laboratory of Molecular Genetics, Department of Immunology, Hellenic Pasteur Institute, 127 Vasilissis Sophias Ave., 11521 Athens, Greece; irinipap@pasteur.gr (I.P.); v.kyrargyri@ucl.ac.uk (V.K.); meuagelidou@gmail.com (M.E.); avoulgarikokota@gmail.com (A.V.-K.); 2 Department of Neuroscience, Physiology & Pharmacology, University College London, Gower Street, London WC1E 6BT, UK; 3 Laboratory of Medical Microbiology, Department of Microbiology, Hellenic Pasteur Institute, 127 Vasilissis Sophias Ave., 11521 Athens, Greece

**Keywords:** neurodegeneration, neuroprotection, neuronal Ca^2+^ signaling, glutamate receptors, NMDA, AMPA receptors, TNF, multiple sclerosis

## Abstract

Mesenchymal stem cells (MSC) provide therapeutic effects in experimental CNS disease models and show promise as cell-based therapies for humans, but their modes of action are not well understood. We previously show that MSC protect rodent neurons against glutamate excitotoxicity in vitro, and in vivo in an epilepsy model. Neuroprotection is associated with reduced NMDA glutamate receptor (NMDAR) subunit expression and neuronal glutamate-induced calcium (Ca^2+^) responses, and increased expression of stem cell-associated genes. Here, to investigate whether MSC-secreted factors modulate neuronal AMPA glutamate receptors (AMPAR) and gene expression, we performed longitudinal studies of enriched mouse cortical neurons treated with MSC conditioned medium (CM). MSC CM did not alter total levels of GluR1 AMPAR subunit in neurons, but its distribution, reducing cell surface levels compared to non-treated neurons. Proportions of NeuN-positive neurons, and of GFAP- and NG2-positive glia, were equal in untreated and MSC CM-treated cultures over time suggesting that neurons, rather than differentially-expanded glia, account for the immature gene profile previously reported in MSC CM-treated cultures. Lastly, MSC CM contained measurable amounts of tumor necrosis factor (TNF) bioactivity and pre-treatment of MSC CM with the TNF inhibitor etanercept reduced its ability to protect neurons. Together these results indicate that MSC-mediated neuroprotection against glutamate excitotoxicity involves reduced NMDAR and GluR1-containing AMPAR function, and TNF-mediated neuroprotection.

## 1. Introduction

Mesenchymal stem cells (MSC) are a heterogeneous population of multipotent progenitor cells that reside in most connective tissues in a perivascular niche and differentiate into cells of mesodermal lineage such as osteoblasts, adipocytes, and chondrocytes [1,2]. They function as cell modulators to stimulate (re)generative processes such as bone repair [3], hematopoiesis, and angiogenesis [4], details of which might be dictated by the environment in which they reside. MSC can be readily isolated from bone marrow and expanded as plastic-adherent cells in culture [5], and therefore are convenient for autologous transplant. The low immunogenicity of MSC in vivo has greatly facilitated experimental studies using autologous, allogenic and xenogenic (human) cells for engraftment or systemic administration in animals, and there is now compelling pre-clinical data to support the application of MSC to the treatment of human neurological conditions [6,7].

Bone marrow-derived MSC provide therapeutic effects in experimental models of human neurological disease, particularly in models of acute disorders such as stroke [8,9,10] and spinal cord injury [11,12], and of immune-mediated diseases such as multiple sclerosis (MS) [13,14,15,16]. Importantly, MSC provide benefit in the CNS when delivered locally or systemically, although the mode of action of systemic MSC and whether they can actively cross the blood brain barrier (BBB) in vivo is not clear. MRI tracking techniques show that systemic MSC rapidly localize to infarcted brain regions in experimental stroke models [17,18], but in a model of epilepsy they exert beneficial effects without significant CNS presence [19]. MSC secrete large amounts of bioactive molecules that mediate immunomodulatory and trophic activities through paracrine mechanisms [20] and might have neuroprotective effects from a peripheral location. Neural effects of MSC include the stimulation of oligodendrocyte [11,21] and neural precursor cells [22], remyelination [11,23], neurogenesis [24], neuroprotection [19,25,26], inhibition of neuroinflammation [10], inflammation-associated oxidative stress [27] and scar formation, and possible regulation of BBB integrity [28]. MSC also have potent immunomodulatory effects and indirectly protect neural tissues by interfering with CNS-directed autoimmune responses in an experimental MS model [13,15].

MSC have strong potential for cell therapy in acute CNS disorders such as stroke and spinal cord injury, and immune-mediated diseases like MS [6]. Early small clinical studies using autologous MSC in patients with MS [29,30,31], cerebral ischemia [32,33], and spinal cord injury [34], reported good tolerability and safety, but large controlled clinical studies are needed to establish efficacy [35]. Based on their strong neuroprotective properties, MSC also hold potential for the treatment of chronic neurodegeneration, for example, in progressive MS. In an open-label phase II study with MSC in patients with secondary progressive MS and evidence for visual path involvement, several measures of efficacy in the anterior visual pathway were met that suggest a neuroprotective effect of MSC [36].

To investigate the mechanism of MSC neuroprotection, we previously show that MSC secreted factors directly protect CNS neurons against glutamate excitotoxicity in vitro and in vivo. Neuroprotection is associated with reduced gene expression of glutamate *N*-Methyl-D-aspartic acid (NMDA) receptor (NMDAR) subunits NR1 and NR2A, as shown in mouse cortical neurons, and Ca^2+^ mobilization in response to glutamate, as shown in rat retinal ganglion cells. Also, short-term (24 h) exposure of mouse cortical neuron cultures to MSC correlates with increased expression of stem cell-associated genes. Since activity-dependent Ca^2+^ influxes through neuronal NMDAR and α-amino-3-hydroxy-5-methyl-4-isoxazolepropionic acid (AMPA) receptors (AMPAR) are important for initiating cell signaling pathways, and transcriptional and post-transcriptional mechanisms that promote dendritic growth, synapse development and neuronal plasticity [37], we hypothesized that MSC neuroprotection might be associated with altered neuronal specialization. To test this hypothesis, here we performed longitudinal studies using mouse cortical neuron cultures to investigate whether exposure to MSC secreted factors alters levels and cellular distribution of neuronal AMPAR, and promotes differential expansion of glial cells, to exclude the possibility that increased glial cell gene expression accounts for the “immature” gene profile previously reported. We also tested whether the pro-inflammatory cytokine tumor necrosis factor (TNF), a known neuroprotective mediator, is involved in MSC neuroprotection.

## 2. Results

### 2.1. MSC Conditioned Medium Protects Isolated Mouse Cortical Neurons against Glutamate Excitotoxicity

**Figure 1:**
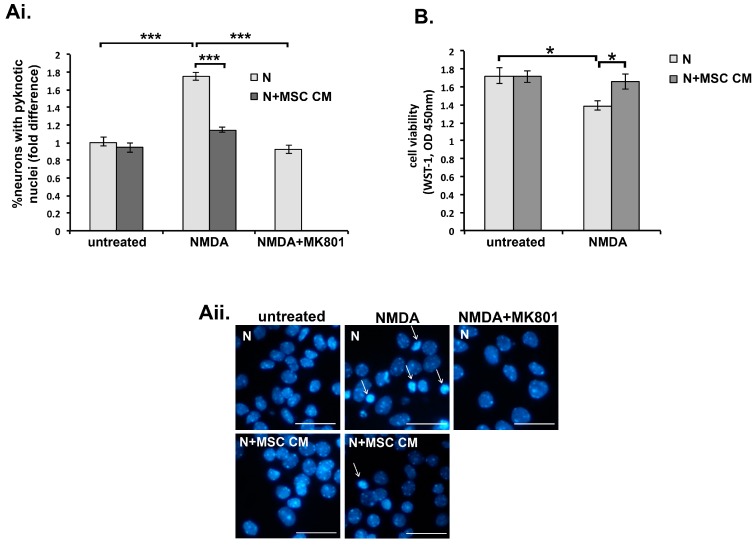
Mesenchymal stem cell (MSC) secreted factors protect cortical neurons against glutamate excitotoxicity. Day 9 in vivo (DIV 9) neurons (N) were pre-treated with medium alone or MSC conditioned medium (MSC CM) (24 h), then left untreated or challenged with glutamate *N*-Methyl-d-aspartic acid (NMDA) (50 μM) supplemented with glycine (10 μM) (24 h), and death was measured at DIV 11 by Hoechst staining (**A**) or the water soluble tetrazolium salt-1 (WST-1) assay (**B**). (**A**) NMDA induced neuronal death, as measured by fold increases in proportions of cells with pyknotic nuclei compared to those in untreated neurons (**i** and **ii**, arrows show pyknotic nuclei). NMDA death was inhibited by pre-treatment of neurons with MSC CM or by presence of 5-Methyl-10,11-dihydro-5H-dibenzo(a,d)cyclohepten-5,10-imine (MK801) (10 μM), a specific NMDA antagonist (**Ai** and **ii**). Neuron death is represented as fold differences compared to measurements in untreated cultures (**Ai**)**.** Representative fields from each culture are shown in the photographs (**Aii**). (**B**) NMDA induced neuronal death, as measured by decreased activity of mitochondrial dehydrogenase using the WST-1 assay, is inhibited by pre-treatment of neurons with MSC CM. Scale bars 50 μM. Results shown are means ± SEM of triplicate samples from one representative of three independent experiments. * *p* < 0.05, *** *p* < 0.001, for pairwise comparisons by Student’s *t*-test.

We first confirmed the neuroprotective properties of conditioned medium harvested from mouse MSC cultures (MSC CM) using a model of glutamate excitotoxicity triggered by NMDAR-triggered Ca^2+^ influx in neurons. Cultured cortical neurons become sensitive to NMDA-induced excitotoxicity from the 10th day in vitro (DIV 10), and sensitivity increases as the neurons mature, resulting in 100% cell death in DIV 19 neurons [38]. In our experiments, neuronal death induced by NMDA (50 μΜ) in DIV 11 neurons ranged between 52–71%, depending on individual cultures. In the primary neuron cultures used here, variability might reflect small differences in cell concentration and/or cellular composition between cultures. We incubated DIV 9 embryonic mouse cortical neuron cultures without or with MSC CM for 24 h (DIV 10) and then exposed them to NMDA (50 μM)/glycine (10 μM) for a further 24 h (DIV 11). As previously reported, MSC CM strongly protected mouse cortical neurons against NMDA-induced excitotoxicity as measured by Hoechst staining and counting of NeuN-positive cells with pyknotic nuclei (Figure 1Ai,Aii), and by preservation of mitochondrial dehydrogenase activity measured using the water soluble tetrazolium salt-1 (WST-1) assay (Figure 1B). As control, treatment of neurons with the NMDAR antagonist 5-Methyl-10,11-dihydro-5H-dibenzo[a,d]cyclohepten-5,10-imine (MK801) fully protected them against NMDA (Figure 1Ai,Aii).

### 2.2. Tumor Necrosis Factor Is Produced by MSC and Contributes to MSC CM-Mediated Neuroprotection

**Figure 2:**
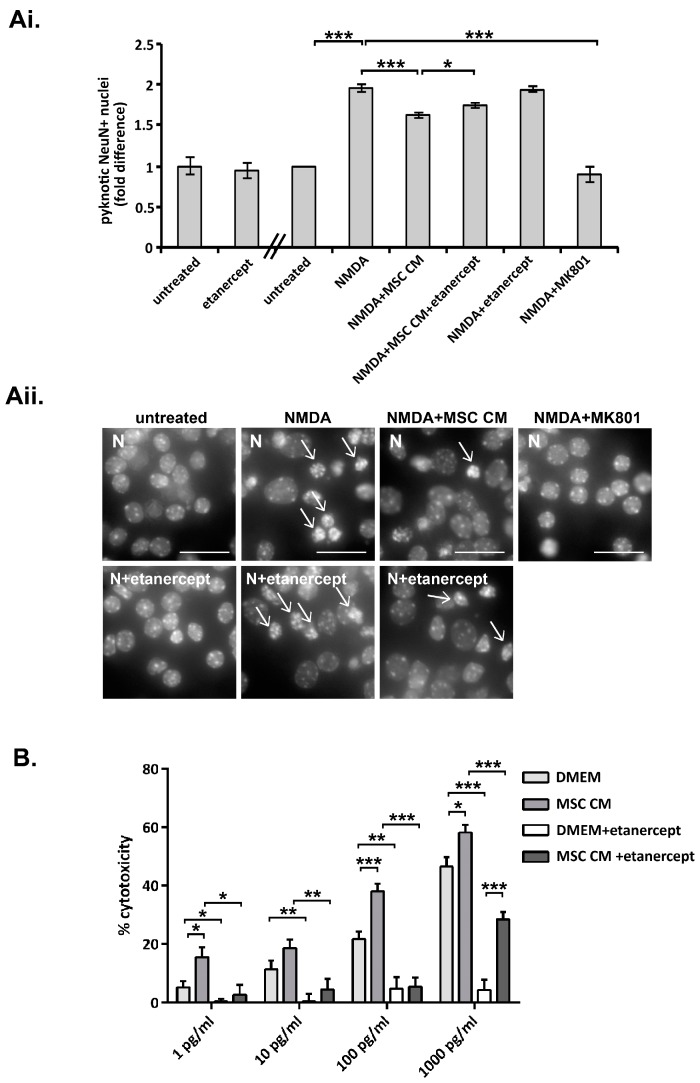
TNF is produced by MSC and contributes to MSC CM-mediated neuroprotection against glutamate excitotoxicity. (**A**) DIV 9 neurons (N) were pre-treated with high glucose DMEM medium alone (hgDMEM) or MSC CM without or with etanercept (100 ng/ml) (24 h), then left untreated or challenged with NMDA (50 μM) supplemented with glycine (10 μM) (24 h), and death was measured at DIV 11 by Hoechst staining. Etanercept treatment consistently resulted in a small but significant increase in NMDA-induced excitotoxicity, as measured by fold changes in proportions of cells with pyknotic nuclei compared to those in cells treated with MSC CM alone (**Ai** and **ii**, arrows show pyknotic nuclei). Neuron death is represented as fold differences compared to measurements in untreated cultures (**Ai**). Representative fields from each culture are shown in the photographs (**Aii**). (**B**) TNF-induced cytotoxicity of L929 fibroblasts was measured in the presence of 1 μg/ml emetine, using increasing amounts of recombinant human TNF (rhTNF) (1, 10, 100 and 1000 pg/ml) added to non-conditioned hgDMEM or MSC CM without or with etanercept (200 ng/ml). MSC CM consistently induced more cytotoxicity than non-conditioned hgDMEM at each concentration of added rhTNF used, demonstrating that MSC CM contains TNF bioactivity (estimated between 0.15–0.24 U/ml). Etanercept inhibited cytotoxicity in all preparations showing that cytotoxicity was specifically induced by TNF. Scale bars 50 μM. Results shown are means ± SEM of triplicate samples from one representative of three independent experiments. * *p* < 0.05, ** *p* < 0.005, *** *p* < 0.001, for pairwise comparisons by Student’s *t*-test.

To investigate whether TNF, a proinflammatory cytokine with strong neuroprotective properties [39], participates in neuroprotection induced by MSC CM, we repeated experiments in the presence of the TNF inhibitor, etanercept. Etanercept is a fusion protein between TNF receptor 2 and the constant chain of immunoglobulin G1 (IgG1) that binds to TNF and acts as a decoy to inhibit its interaction with endogenous TNF receptors [40]. Incubation of DIV 9 neurons with normal medium containing etanercept (100 ng/ml) for 24 h (DIV 10) did not alter the proportion of neurons with pyknotic nuclei following challenge or not with NMDA for 24 h (DIV 11) (Figure 2A). However, incubation of neurons with MSC CM containing etanercept consistently resulted in a small but significant reduction in neuroprotection against NMDA excitotoxicity (Figure 2A). These results indicate that TNF plays a role in MSC CM-mediated neuroprotection.

To confirm that MSC produce TNF we measured TNF bioactivity in MSC CM using the L929 cytotoxicity assay. Incubation of L929 fibroblasts with TNF, in the presence of emetine to block de novo protein synthesis and the activation of cell survival pathways, results in TNF cytotoxicity. TNF levels in MSC CM were below the limits of detection using this assay. Therefore, increasing known amounts of recombinant human TNF (rhTNF) (1–1000 pg/ml), were added to both non-conditioned medium (control) and MSC CM and the levels of TNF cytotoxicity were compared at each TNF concentration. TNF induced a dose dependent increase in cell cytotoxicity when added to both non-conditioned DMEM and MSC CM (Figure 2B). However, TNF cytotoxicity was consistently higher with MSC CM, thereby demonstrating that MSC CM contains TNF bioactivity (ranging between 0.15–0.24 U/ml TNF in different preparations). Addition of the TNF inhibitor etanercept (200 ng/ml) completely inhibited cytotoxicity by non-conditioned DMEM at all concentrations of added rhTNF (1–1000 ng/ml), and by MSC CM at concentrations of added rhTNF up to 1000 ng/ml, where inhibition was significant but partial, thereby further confirming the presence of TNF bioactivity in MSC CM (Figure 2B).

### 2.3. MSC Secreted Factors Do Not Induce Differential Survival of Neurons or Expansion of Non-Neuronal Cells in Long-Term Cultures

**Figure 3:**
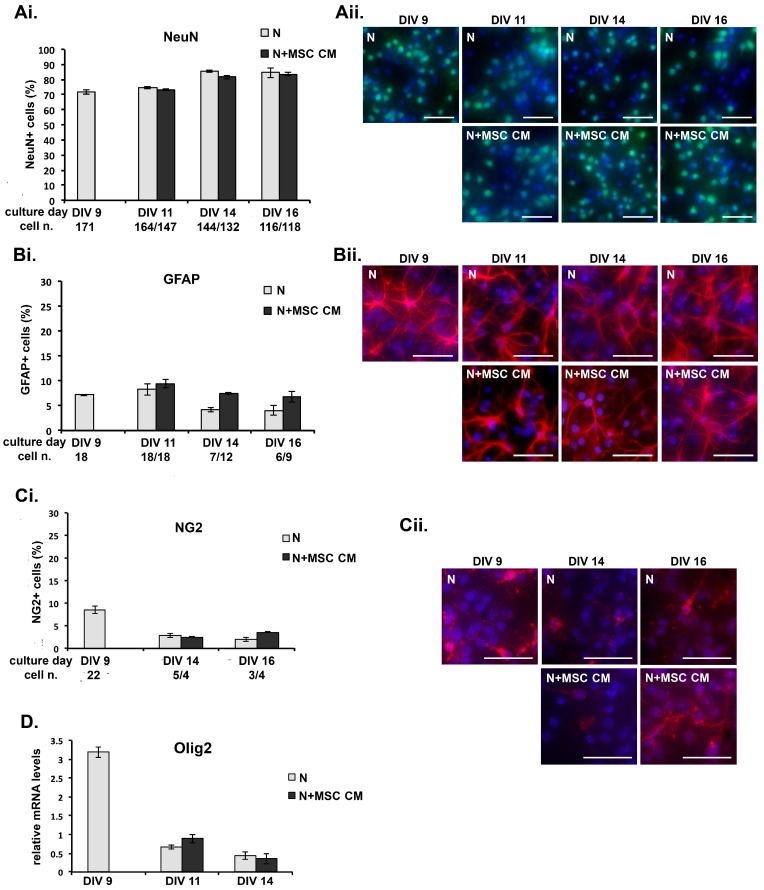
MSC secreted factors do not induce differential survival of neurons or expansion of non-neuronal cells in long-term cultures. (**A**–**C**) DIV 9 cortical neurons were grown in MSC CM for different lengths of time, fixed and immunostained using antibodies for cell markers, (**A**) neuronal nuclei (NeuN) for neurons, (**B**) glial fibrillary acidic protein (GFAP) for mature astrocytes and new neurons, and (**C**) neural/glial antigen 2 (NG2) for oligodendrocyte precursor cells (OPC), microglia/macrophages and pericytes, and nuclei counterstained with the fluorescent DNA stain 4′,6-diamidino-2-phenylindole (DAPI). The proportions of each cell type (percentage of specifically-immunostained cells compared to total numbers of DAPI-stained cells) in the untreated and MSC CM-treated cultures at each time point are shown (**Ai**, **Bi**, **Ci**). The total numbers of DAPI-stained cells/field in cultures at each time point and condition are shown under the *x*-axis. Representative fields from each culture are shown in photographs (**Aii**, **Bii**, **Cii**). Scale bars 100 μM. (**D**) DIV 9 cortical neurons were grown in MSC CM for different lengths of time, and levels of mRNA for Olig2 measured by quantitative PCR. Results shown are means ± SEM of triplicate samples from one representative of three independent experiments. *p* > 0.05 for pairwise comparisons by Student’s *t*-test.

To exclude the possibility that exposure of mouse cortical neurons to MSC CM favors the differential survival of neurons or glial cells that might affect the gene expression pattern of the cultures, we performed longitudinal studies to compare the growth of different cell types in control and MSC CM-treated neuron cultures. Neurons were grown on coverslips and immunostained at different culture time points using cell lineage marker antibodies, including anti-neuronal nuclei (NeuN) for post-mitotic neurons, anti-GFAP for mature astrocytes and neural precursor cells (NPC), and anti-NG2 chondroitin sulfate proteoglycan which is expressed by oligodendrocyte precursor cells (OPC), microglia/macrophages and pericytes [41]. While enriched cultures of cortical neurons were used for all of our experiments, other non-neuronal cells are also present at DIV 9 after Ara C treatment, including GFAP- (6–9%) and NG2-positive cells (5–10%) (Figure 3A–C). MSC CM was added to the neuronal culture on DIV 9 and cells were fixed at different time points (DIV 9, DIV 11, DIV 14, DIV 16), immunostained using the cell marker antibodies, counterstained with DAPI and counted. Total numbers of NeuN-positive neurons, GFAP-positive astrocytes and NG2-positive cells decreased equally in control and MSC CM-treated cultures over time (Figure 3A–C). However, neither the gross morphology nor the proportion of neurons in the cultures, as measured by the percentage of NeuN-positive cells relative to total numbers of DAPI-stained cells, was altered by incubation with MSC CM (Figure 3A). Similarly, MSC CM did not alter the representation of astrocytes or NG2-positive cells in the cultures at any time point, as measured by the percentages of GFAP- (Figure 3B) and NG2-positive cells (Figure 3C), respectively.

In some cultures, cells were used for mRNA isolation and measurement of the expression of Olig2, which encodes a transcription factor involved in the suppression of astrocyte formation and the development of early oligodendrocyte progenitors and certain types of neurons from precursor cells. Consistent with the results from cell counting, levels of mRNA transcripts for Olig2 were equal in untreated and MSC CM-treated cultures at each time point (Figure 3D). These results indicate that MSC do not promote differential survival of neurons or glial cells in long-term cultures of mouse cortical neurons, and suggest that MSC CM-induced changes in gene expression previously reported in short-term cultures [19] reflect cell autonomous changes in neurons.

### 2.4. MSC CM Prevents Increase in Ca^2+^ Levels in Mouse Cortical Neurons in Response to Glutamate Receptor Stimulation

**Figure 4:**
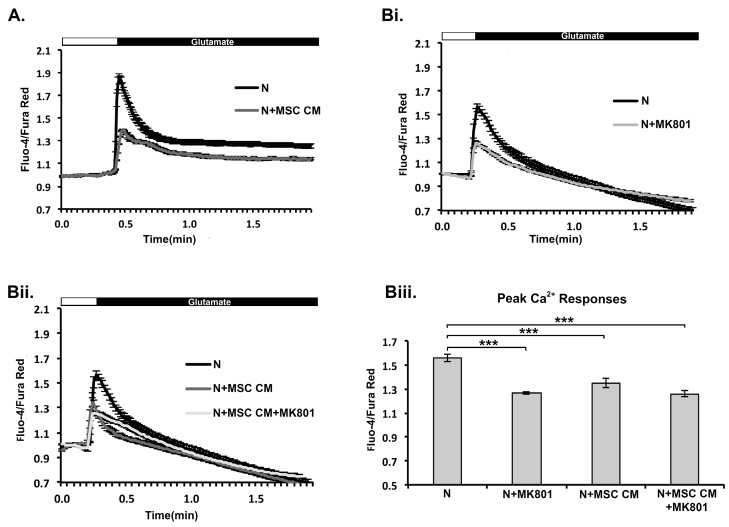
MSC CM-mediated neuroprotection in mouse cortical neurons is associated with reduced glutamate-induced Ca^2+^ responses. DIV 9 neurons (N) were pre-treated with MSC CM (24 h) and glutamate-induced intracellular Ca^2+^ levels were measured by Fluo-4 and Fura Red imaging. Perfusion of neurons with glutamate/glycine transiently increased Ca^2+^ levels in control cells, while responses were reduced in MSC CM-treated cells (**A**). Addition of MK801 inhibited Ca^2+^ responses in untreated (**Bi** and **Biii**), but not MSC CM-treated (**Bii** and **Biii**) cells showing that the effects of MSC CM were mediated through the NMDAR. Peak responses in Bi and Bii are also represented as a plot (**Biii**). Results shown are means ± SEM of triplicate samples from one representative of three independent experiments. *** *p* < 0.001, pairwise comparison by Student’s *t*-test (**A**), and multiple comparisons by one-way ANOVA with Bonferroni test (**B**).

In a previous study we show that MSC CM protects rat retinal ganglion cells (RGC) against glutamate excitotoxicity, and that protection is associated with prevention of increases in intracellular Ca^2+^ levels in response to glutamate receptor stimulation, mainly through NMDA receptors [19]. Here we investigated whether MSC CM-mediated protection of mouse cortical neurons involves a similar mechanism. We repeated these experiments to avoid misinterpretations arising from differences in species (rats/mice) or neuronal populations (retinal ganglion cells/cortical neurons). DIV 9 neurons were incubated for 24 h with MSC CM and changes in Ca^2+^ levels in response to glutamate were measured by Fluo-4/Fura Red imaging. As in rat RGC, Ca^2+^ mobilization was markedly reduced in MSC CM-treated compared to untreated mouse cortical neurons, as measured by decrease in Fluo-4/Fura Red ratio (Figure 4A,B). Glutamate-induced Ca^2+^ responses in untreated neurons were markedly inhibited by the NMDA inhibitor MK801, showing NMDA receptor dependence (Figure 4B). Glutamate-induced increases in intracellular Ca^2+^ levels in MSC CM-treated neurons were also markedly inhibited, and addition of MK801 did not induce further inhibition (Figure 4B). These results suggest that MSC CM protect mouse cortical neurons against glutamate excitotoxicity by reducing glutamate-induced Ca^2+^ responses and this latter effect is dependent on NMDA receptor function. 

### 2.5. MSC CM Reduces Cell Surface Expression of the GluR1 Subunit of the AMPAR on Cortical Neurons

**Figure 5:**
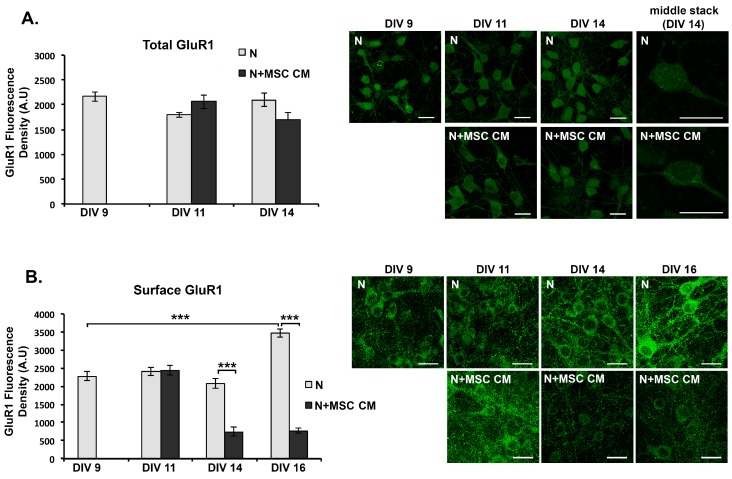

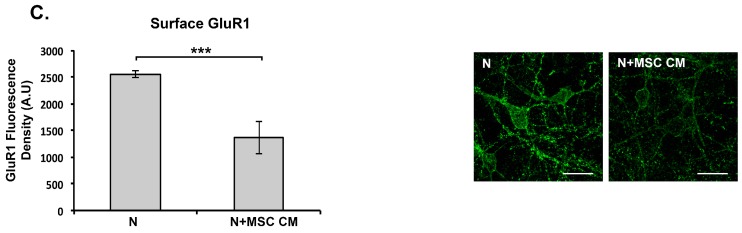
MSC secreted factors reduce neuronal cell surface, but not total, levels of the GluR1 α-amino-3-hydroxy-5-methyl-4-isoxazolepropionic acid receptor (AMPAR) subunit in mouse cortical neuron (N) cultures. DIV 9 neurons were grown in MSC CM for different lengths of time, fixed and immunostained using anti-GluR1-NT antibody. (**A**) Total neuronal levels of GluR1 (surface and intracellular) were measured in fixed, Triton X-100-permeabilized neurons after different times of culture with MSC CM or control medium. (**B**) Cell surface levels of GluR1 were measured in paraformaldehyde/sucrose fixed neurons after different times of culture with MSC CM or control medium. (**C**) Cell surface levels of GluR1 were measured in live neurons by immunostaining with anti-GluR1-NT antibody after 24 h culture with MSC CM or control medium (DIV 9) prior to fixation. Representative fields from each culture are shown in photographs including individual stacks from DIV 14 neurons showing differential localization of GluR1 in untreated and MSC CM-treated neurons (**A**). Scale bars 20 μM. Results shown are means ± SEM of triplicate samples from one representative of three independent experiments. *** *p* < 0.001 for indicated pairwise comparisons by Student’s *t*-test.

We next investigated whether MSC CM-mediated blockade of NMDAR-dependent Ca^2+^ responses in mouse neurons are associated with altered cell surface levels of AMPAR, which are known to be regulated by Ca^2+^ signaling. Detection of the GluR1 subunit of the AMPAR was performed by immunocytochemistry using an antibody to GluR1 N-terminus (clone RH95) in 3 different staining procedures. First, we measured the total levels of GluR1 (surface and intracellular) in paraformaldehyde-fixed, Triton X-100-permeabilised neurons after different times of culture with MSC CM. MSC CM did not alter total GluR1 levels compared to untreated neurons at any time point studied (Figure 5A). However, when we observed individual stacks from the confocal fluorescent microscope we noticed that GluR1 showed different subcellular localization in the untreated- and MSC CM-treated cells. In MSC CM-treated cells, GluR1-immunoreactivity was preferentially localized to perinuclear structures, while in untreated cells it appeared more evenly dispersed throughout the cytoplasm (Figure 5A).

To measure cell surface levels of GluR1, we used paraformaldehyde/sucrose-fixed, non-permeabilised DIV 9 neurons for immunocytochemistry. Untreated neurons showed a significant increase in cell surface GluR1 at DIV 16 compared to DIV 9 (Figure 5B). In contrast, MSC CM-treated neurons showed progressive reduction of surface levels of GluR1-immunoreactivity over time, with significantly lower levels compared to untreated neurons at DIV 14 and DIV 16 in culture (Figure 5B). To confirm this finding we also measured GluR1 levels with a more sensitive method by immunostaining live DIV 9 neurons, which were untreated or treated for 24 h (DIV 10) with MSC CM, with anti GluR1 antibody before fixation. Under these conditions, MSC CM reduced surface levels of GluR1-immunoreactivity compared to those in untreated cells (Figure 5C). Taken together, these results suggest that MSC secrete factors that decrease cell surface levels of the AMPAR GluR1 subunit in mouse cortical neurons, probably as a result of reduced NMDAR-dependent Ca^2+^ signaling. 

## 3. Discussion

Understanding of the mechanisms by which MSC protect CNS neurons against neurodegeneration is important in view of the promise these cells show as a cell-based therapy for neurological disorders. MSC have strong immune modulatory and trophic effects on non-neuronal cells that indirectly mediate neuroprotection via bystander mechanisms [6]. They also mediate direct neuroprotective effects but there is relatively little information concerning the mechanisms of action. In vitro, bone marrow MSC CM protects primary rat hippocampal neurons against staurosporine- and amyloid beta-induced apoptosis through activation of the phosphatidylinositide 3-kinase/protein kinase B (PI3-K/Akt) signaling pathway, which is a critical cellular survival pathway [26]. Also, human bone marrow MSC CM protects primary rat cortical neurons against death induced by trophic factor withdrawal or nitric oxide (NO) through activation of the PI3-K/Akt pathway, and protection is partially mediated by MSC-derived brain-derived neurotrophic factor [25]. In a previous study we showed that bone marrow MSC secreted factors protect primary mouse cortical neurons and rat retinal ganglion neurons against glutamate excitotoxicity [19]. Glutamate excitotoxicity is known to damage neurons and oligodendrocytes in a variety of neurodegenerative conditions, including experimental autoimmune encephalomyelitis (EAE) and MS, and therefore represents a valid model for investigating the neuroprotective effects of MSC [42,43,44,45]. Protection is associated with reduced expression of NMDAR subunits and glutamate-induced Ca^2+^ responses, as well as increased expression of genes associated with non-neuronal and stem cell types, as determined by transcriptome analysis of MSC CM-treated neuron cultures [19]. Here, we show that MSC CM reduces levels of the GluR1 AMPAR subunit on the neuronal cell surface, thereby further reducing neuronal glutamate signaling. We also provide evidence that the “immature” gene expression signature previously associated with neuroprotection in short-term cultures reflects cell autonomous changes in neurons, and therefore might be a direct result of reduced AMPAR signaling. We further reveal a minor role for TNF in MSC neuroprotection.

There is a major lack of understanding of how systemically administered MSC mediate neuroprotective functions within the CNS in order to evaluate the in vivo relevance of our findings. One possibility is that they migrate across the BBB but it is controversial whether systemic MSC can enter the CNS, and if so under which conditions. A second possibility is that MSC secreted factors mediate neuroprotection through paracrine mechanisms. Several molecules have been identified. In one study, systemic MSC CM promoted functional recovery in EAE through hepatocyte growth factor (HGF) and its main receptor, the proto-oncogene protein cMet, effects that were attributed to immunosuppression and promotion of remyelination [46]. In other studies, fibroblast growth factor (FGF)-II [47], and BDNF [25] provided neuroprotection. Here we identify, for the first time, TNF as a neuroprotective mediator present in MSC CM. In vitro studies by several groups including our own, have established that TNF pretreatment directly protects neurons against a wide range of toxic stimuli by signaling through cell surface TNF receptors 1 and 2 and activation of pro-survival gene transcription pathways [39,48,49]. A third possibility is that systemic MSC mediate neuroprotection by activating endogenous CNS cells. NPC promote repair in MS models [50] and protect neurons against glutamate excitotoxicity [51], while NG2^+^ cells including OPC, microglia and pericytes enhance axon regeneration after spinal cord injury [52]. There is also recent evidence that pericytes, which are localized perivascularly, give rise to MSC in vitro and MSC-like cells in vivo [53], opening the intriguing possibility that the CNS contains an endogenous population of MSC-like cells that might have direct beneficial effects under conditions of injury and disease. MSC promote adult NPC differentiation towards the oligodendroglial lineage in rat hippocampal slice cultures [22,23], and MSC CM promote oligodendroglial differentiation under astrocyte endorsing conditions in OPC cultures [21] suggesting they might support myelin repair.

Our finding that MSC secreted factors prevent NMDA-induced expression of NMDAR subunits NR1 and NR2A [19] and, as we show here, glutamate-induced Ca^2+^ responses and surface GluR1 in cultured mouse cortical neurons, suggests that MSC protect neurons by downregulating the basic components of glutamatergic neurotransmission. Most of the excitatory neurons in the CNS are glutamatergic and such a mechanism would be expected to have important consequences in vivo. One of the main results of ligand binding to Ca^2+^-permeable NMDAR and AMPAR on the surface of glutamatergic neurons is a rapid and transient rise in intracellular Ca^2+^. Calcium then acts as a second messenger to stimulate several intracellular signaling pathways, including post-transcriptional mechanisms that lead to insertion and removal of AMPAR from the surface of dendrites, and gene transcription programs that promote dendritic growth, synapse development and neuronal plasticity [37]. Under physiological conditions, such mechanisms are believed to regulate the strength or efficiency of synaptic transmission and to underlie processes of memory and learning [54], but under conditions of disease and injury, excess glutamate induces acute excitotoxicity leading to neuronal damage and necrosis [45,55]. It remains to be determined whether the effects of MSC on neuronal glutamate receptors observed in vitro have relevance in vivo, by promoting neuroprotection against acute excitotoxicity under pathological conditions, but also possibly affecting neuronal processes such as synaptic plasticity under physiological conditions.

## 4. Materials and Methods

### 4.1. Animals

From 6- to 8-week-old female C57BL/6 mice were used for the isolation of bone marrow MSC and day in utero 14.5 C57BL/6 embryos (E14.5) for the isolation of cortical neurons. Animals were maintained under specific pathogen-free conditions at the animal facilities of the Hellenic Pasteur Institute and all experimental procedures were approved by national authorities and conformed to Animal Research: Reporting of In Vivo Experiments (ARRIVE) guidelines and European Union Directive 2010/63/EU for animal experiments.

### 4.2. Cell Culture and Experimental Treatments

MSC were isolated from the bone marrow of C57BL/6 mice using plastic adherence capacity as previously described [5]. MSC were used for in vitro and in vivo experiments after the 6th passage when the culture contained >98% CD11b^−^Sca1^+^CD44^+^MSC as assessed by FACS analysis using antibodies to the surface markers Sca1 (clone E13-161.7), CD44 (clone IM7) and CD11b (clone M1/70) (BD Biosciences, San Jose, NJ, USA) and a FACSCalibur with CellQuest software (BD Biosciences, NJ, USA) [19]. Dissociated neocortical cell cultures were prepared from E14.5 mice as previously described and grown in 48-well culture plates in high-glucose DMEM containing 5% horse serum, 5% fetal bovine serum (FBS), and 2mM glutamine (hgDMEM) [19]. At day 6 in vitro (DIV 6), non-neuronal cell division was halted by addition of 10 μM arabinose-C (AraC) (Sigma, St. Louis, MO, USA) for 3 days, and neurons were used for experiments after DIV 9 in the absence of AraC. DIV 9 neurons (4 × 10^5^/cm^2^) were pre-treated with MSC CM for 24 h (DIV 10) or for 2, 5 and 7 days (DIV 11, DIV 15, DIV 16, respectively). MSC CM was prepared by culturing MSC (2 × 10^4^/cm^2^) in hgDMEM for 24 h and was used to replace 75% of the neuronal culture medium. Excitotoxic death was induced in DIV 10 neurons by exposure to 50 μM NMDA (Sigma-Aldrich, St. Louis, MO, USA; M3262) supplemented with 10 μM glycine for 24 h. The NMDAR antagonist MK801 was added to culture medium at 10 μM. In TNF neutralization experiments etanercept (Amgen, Thousand Oaks, CA, USA; 100 ng/mL) [40], an inhibitor of both soluble and transmembrane forms of TNF, was added to MSC CM for 30 min at 37 °C prior to addition to the neuron cultures.

### 4.3. TNF Bioactivity and Cell Death Assays

Neuronal death was calculated on DIV 11 as a percentage of Hoechst 33342-stained pyknotic nuclei in total numbers of neurons and shown as the mean % ± SEM from photographs of 10–15 randomly selected fields, containing from 50 to 100 cells/field, taken at magnification ×40. Values were normalized against measurements in untreated cultures. Pyknotic nuclei are characteristic of neuronal necrosis induced by acute excitotoxicity in vivo and in vitro [55]. Cell viability was also measured using the WST-1 assay (Roche, ‎Basel, Switzerland), which measures cell metabolism enzymes (mitochondrial dehydrogenases) that reduce the water-soluble tetrazole of WST-1 dye by converting it to insoluble formazan to give a measurable color. WST-1 assay reagent was added to neuronal cultures, grown on 96-well plated and treated as described above, at a concentration of 10% according to manufacturer’s instructions. Cells were incubated for 1 h (37 °C) and optical density (OD) of the medium read at 450nm and 630nm on an ELISA plate reader (Dynex Technologies, Chantilly, VA, USA). TNF bioactivity in MSC CM was measured by the L929 cytotoxicity assay using emetine dihydrochloride (Sigma, St. Louis, MO, USA)-treated mouse L929 fibroblasts as previously described [56]. Briefly, 2.5 × 10^5^/ml cells were seeded on flat bottomed 96-well plates (100 μl/well) in DMEM supplemented with 5% FBS for 20–24 h. Then medium was replaced by hgDMEM or MSC CM and various concentrations of rhTNF (R&D, Minneapolis, MN, USA) (1–1000 pg/ml) were added per well in the presence of emetine (1 μg/ml). Death was inhibited by TNF inhibitor etanercept (200 ng/ml). Every condition was represented with five-plicates. After a 20 h incubation period, cell viability was determined by crystal violet staining and plates read at 570 nm on an ELISA plate reader. The percent cytotoxicity was calculated from the formula 100(*a – b*)/*a*, where *a* and *b* are the mean absorbances of five-plicate wells with medium alone and test sample, respectively. 1U of TNF bioactivity was taken as the amount of activity inducing 50% cytotoxicity. 

### 4.4. Immunocytochemistry

Neurons were grown on plastic coverslips, coated with poly-d-lysine, adapted in 48-well culture plates in hgDMEM, and on DIV 9 medium was replaced to 75% with MSC-CM or control media. Neurons were cultured in the absence or presence of MSC CM and fixed at different time points of culture with 4% paraformaldehyde in PBS pH 7.4 on ice for 20 min. Cells were treated with blocking buffer (10% FBS, 0,3% Triton X-100 in PBS) and immunostained with mouse anti-NeuN monoclonal antibody (1/300; Millipore, Princeton, NJ, USA), rabbit anti-GFAP polyclonal antibody (1/400; DAKO), rabbit anti-NG2 polyclonal antibody (1/200; Millipore), or mouse anti-glutamate receptor (AMPAR) 1-N terminus (GluR1-NT) monoclonal antibody (clone RH95) (1/50; Millipore), followed by either goat anti-mouse (1/1000; Molecular Probes) or anti-rabbit Alexa Fluor 568 (1/1000; Molecular Probes, Invitrogen, MA, USA) antibody. Nuclei were stained with DAPI. To measure levels of cell surface GluR1, neurons were fixed with 4% paraformaldehyde + 4% sucrose in PBS pH 7.4 and blocking buffer without Triton X-100 before staining. Alternatively, live neurons were stained using anti-GluR1-NT monoclonal antibody (1/50; Millipore), fixed with 4% paraformaldehyde + 4% sucrose in PBS pH 7.4 and incubated with Alexa-488-labeled second goat anti-mouse antibody (green) (1/1000; Molecular Probes). The proportion of immunostained cells in each culture condition was calculated as a percentage of normal-appearing DAPI stained nuclei and shown as the mean % ± SEM from photographs of 10–15 randomly selected fields, containing 80 to 400 cells/ field, taken at magnifications ×10 for NeuN and ×20 for the other stainings. The fluorescence density (positive pixels) from GluR1 staining was measured using the software Image Pro from 10–20 individual neurons from each random field. Values were normalized to the cell area. Cell^A imaging software (Olympus, Japan) was used for analysis.

### 4.5. Calcium Imaging

DIV 9 neurons grown in 48-well culture plates (4 × 10^5^/cm^2^) were incubated without or with MSC CM for 24 h and further treated without or with 100 μM NMDA for 12 h, which has previously been shown to increase NMDAR expression [19]. Cells were briefly washed in oxygenated Hank’s buffered salt solution buffer pH 7.4, containing 2% HEPES buffer, 1 mM CaCl_2_ and 1 mM MgSO_4_ at RT (imaging buffer), and loaded with fluorescent Ca^2+^-sensitive dyes by incubation in imaging buffer containing 5 μΜ Fura Red AM and 5 μΜ Fluo-4 AM (Invitrogen, Massachusetts ,USA) for 30 min. The combined use of Fluo-4 (or Fluo-3) and Fura Red allows dual-emission ratiometric confocal measurements using visible light excitation [57]. Fluo-4 (emission 488 nm) shows increase in fluorescence upon a rise in intracellular Ca^2+^ concentrations, while Fura Red (emission 637 nm) fluorescence decreases so that increases in intracellular Ca^2+^ concentrations are measured as increases in Fluo-4/Fura Red ratios. After dye-loading, cells were placed into fresh imaging buffer and equilibrated in the microscope chamber at 5% CO_2_, 70–75% humidity, 37 °C for 30 min for the experiment. In some wells, MK801 (10 μM) was added to the culture 30 min before glutamate challenge. Neurons were imaged using a fluorescent microscope, Olympus Time lapse ΙX81 Cell-R with excitation at 488 nm, and emission was measured at 520 nm for Fluo-4 and 650 nm for Fura Red. A video recorded fluorescence levels before (baseline) (~0.5 min), and after the addition of 200 μΜ glutamate/10 μΜ glycine (~2 min) to the experimental solution. Repeated images of each field, containing ~80 cells/field, were taken every 490 msec for the duration of the experiment. From each field, ~20–30 cells (regions of interest—ROIs) were selected for analysis of Ca^2+^ responses using Cell^R (Olympus, Japan) software. Ratiometric values (Fluo-4/Fura Red) were calculated before and after glutamate stimulus. Glutamate-induced ratios were normalized against baseline ratios, and results are expressed as means of the selected ROIs.

### 4.6. Real-Time Quantitative RT-PCR

Total RNA was isolated from neurons cultured in the absence or presence of MSC-CM at DIV 9, DIV11, DIV 14 and DIV 16. Quantitative RT-PCR was performed using QuantiFastTM SYBR^®^ green RT-PCR kit (Qiagen Inc., Hilden, Germany) and a QuantiTect Primer Assay for oligodendrocyte lineage transcription factor, *Olig2* (QT01041089) (Qiagen Inc., Germany), according to the manufacturer's instructions. Normalization was done using *Gusb* (QT00176715) (Qiagen Inc., Germany). All reactions were performed using the LightCycler system (Roche, Penzberg, Germany). At the end of each PCR run, melting curve analysis was also performed to verify the integrity and homogeneity of PCR products. Gene expression levels were calculated using standard curves for each gene. Standard curves were created by plotting threshold cycle (CT) values versus the logarithm of serially diluted amounts of total RNA isolated from untreated DIV 9 neuron cultures. Least squares methods were used for the determination of A and B values in the equation CT = A × Log·(CRNA) + B. The coefficient of determination (R2) was greater than 0.99.

### 4.7. Statistics

Statistical analyses were performed using Graphpad Prism 7. Data are presented as mean ± SEM. Student’s *t*-test or Mann-Whitney was performed for pairwise comparisons between neuron viabilities as measured by Hoechst staining, proportions of immunoreactive cells, relative mRNA levels and cell cytotoxicity in the L929 and WST-1 assays. One-way ANOVA was performed for comparisons of NMDA-induced intracellular Ca^2+^ levels. Values (*) of *p* ≤ 0.05 were considered statistically significant.

## Abbreviations

AMPA	α-amino-3-hydroxy-5-methyl-4-isoxazolepropionic acid
AMPAR	AMPA receptors
Ara C	Arabinose C
BBB	Blood brain barrier
Ca^2+^	Calcium
CNS	Central nervous system
DAPI	4′-6-diamidino-2-phenylindole
DMEM	Dulbecco’s Modified Eagle Medium
EAE	Experimental autoimmune encephalomyelitis
FBS	Fetal bovine serum
FGF-II	Fibroblast growth factor-II
GluR1	AMPAR subunit
hgDMEM	High glucose DMEM
MSC	Mesenchymal stem cells
MSC CM	Mesenchymal stem cells conditioned medium
NeuN	Neuronal nuclei
NPC	Neural precursor cells
NG2	Neural/glial antigen 2
NMDA	*N*-Methyl-d-aspartic acid
NMDAR	NMDA receptor
RGC	Retinal ganglion cells
TNF	Tumor necrosis factor
